# Biomarker Profiling by Nuclear Magnetic Resonance Spectroscopy for the Prediction of All-Cause Mortality: An Observational Study of 17,345 Persons

**DOI:** 10.1371/journal.pmed.1001606

**Published:** 2014-02-25

**Authors:** Krista Fischer, Johannes Kettunen, Peter Würtz, Toomas Haller, Aki S. Havulinna, Antti J. Kangas, Pasi Soininen, Tõnu Esko, Mari-Liis Tammesoo, Reedik Mägi, Steven Smit, Aarno Palotie, Samuli Ripatti, Veikko Salomaa, Mika Ala-Korpela, Markus Perola, Andres Metspalu

**Affiliations:** 1 The Estonian Genome Center, University of Tartu, Tartu, Estonia; 2 Institute for Molecular Medicine Finland, University of Helsinki, Helsinki, Finland; 3 Department of Chronic Disease Prevention, National Institute for Health and Welfare, Helsinki, Finland; 4 Computational Medicine, Institute of Health Sciences, University of Oulu and Oulu University Hospital, Oulu, Finland; 5 NMR Metabolomics Laboratory, School of Pharmacy, University of Eastern Finland, Kuopio, Finland; 6 The Broad Institute of MIT and Harvard, Cambridge, Massachusetts, United States of America; 7 Division of Genetics, Children's Hospital, Boston, Massachusetts, United States of America; 8 Division of Endocrinology, Children's Hospital, Boston, Massachusetts, United States of America; 9 Program in Genomics, Children's Hospital, Boston, Massachusetts, United States of America; 10 Department of Genetics, Harvard Medical School, Boston, Massachusetts, United States of America; 11 Wellcome Trust Sanger Institute, Hinxton, United Kingdom; 12 Computational Medicine, School of Social and Community Medicine, University of Bristol, Bristol, United Kingdom; 13 Institute of Molecular and Cell Biology, University of Tartu, Tartu, Estonia; Imperial College London, United Kingdom

## Abstract

**Background:**

Early identification of ambulatory persons at high short-term risk of death could benefit targeted prevention. To identify biomarkers for all-cause mortality and enhance risk prediction, we conducted high-throughput profiling of blood specimens in two large population-based cohorts.

**Methods and Findings:**

106 candidate biomarkers were quantified by nuclear magnetic resonance spectroscopy of non-fasting plasma samples from a random subset of the Estonian Biobank (*n* = 9,842; age range 18–103 y; 508 deaths during a median of 5.4 y of follow-up). Biomarkers for all-cause mortality were examined using stepwise proportional hazards models. Significant biomarkers were validated and incremental predictive utility assessed in a population-based cohort from Finland (*n* = 7,503; 176 deaths during 5 y of follow-up). Four circulating biomarkers predicted the risk of all-cause mortality among participants from the Estonian Biobank after adjusting for conventional risk factors: alpha-1-acid glycoprotein (hazard ratio [HR] 1.67 per 1–standard deviation increment, 95% CI 1.53–1.82, *p* = 5×10^−31^), albumin (HR 0.70, 95% CI 0.65–0.76, *p* = 2×10^−18^), very-low-density lipoprotein particle size (HR 0.69, 95% CI 0.62–0.77, *p* = 3×10^−12^), and citrate (HR 1.33, 95% CI 1.21–1.45, *p* = 5×10^−10^). All four biomarkers were predictive of cardiovascular mortality, as well as death from cancer and other nonvascular diseases. One in five participants in the Estonian Biobank cohort with a biomarker summary score within the highest percentile died during the first year of follow-up, indicating prominent systemic reflections of frailty. The biomarker associations all replicated in the Finnish validation cohort. Including the four biomarkers in a risk prediction score improved risk assessment for 5-y mortality (increase in C-statistics 0.031, *p* = 0.01; continuous reclassification improvement 26.3%, *p* = 0.001).

**Conclusions:**

Biomarker associations with cardiovascular, nonvascular, and cancer mortality suggest novel systemic connectivities across seemingly disparate morbidities. The biomarker profiling improved prediction of the short-term risk of death from all causes above established risk factors. Further investigations are needed to clarify the biological mechanisms and the utility of these biomarkers for guiding screening and prevention.

*Please see later in the article for the Editors' Summary*

## Abstract 2

In this study, Würtz and colleagues conducted high-throughput profiling of blood specimens in two large population-based cohorts in order to identify biomarkers for all-cause mortality and enhance risk prediction. The authors found that biomarker profiling improved prediction of the short-term risk of death from all causes above established risk factors. However, further investigations are needed to clarify the biological mechanisms and the utility of these biomarkers to guide screening and prevention.

*Please see later in the article for the Editors' Summary*

## Editors' Summary

**Background:**

A biomarker is a biological molecule found in blood, body fluids, or tissues that may signal an abnormal process, a condition, or a disease. The level of a particular biomarker may indicate a patient's risk of disease, or likely response to a treatment. For example, cholesterol levels are measured to assess the risk of heart disease. Most current biomarkers are used to test an individual's risk of developing a specific condition. There are none that accurately assess whether a person is at risk of ill health generally, or likely to die soon from a disease. Early and accurate identification of people who appear healthy but in fact have an underlying serious illness would provide valuable opportunities for preventative treatment.

While most tests measure the levels of a specific biomarker, there are some technologies that allow blood samples to be screened for a wide range of biomarkers. These include nuclear magnetic resonance (NMR) spectroscopy and mass spectrometry. These tools have the potential to be used to screen the general population for a range of different biomarkers.

**Why Was This Study Done?:**

Identifying new biomarkers that provide insight into the risk of death from all causes could be an important step in linking different diseases and assessing patient risk. The authors in this study screened patient samples using NMR spectroscopy for biomarkers that accurately predict the risk of death particularly amongst the general population, rather than amongst people already known to be ill.

**What Did the Researchers Do and Find?:**

The researchers studied two large groups of people, one in Estonia and one in Finland. Both countries have set up health registries that collect and store blood samples and health records over many years. The registries include large numbers of people who are representative of the wider population.

The researchers first tested blood samples from a representative subset of the Estonian group, testing 9,842 samples in total. They looked at 106 different biomarkers in each sample using NMR spectroscopy. They also looked at the health records of this group and found that 508 people died during the follow-up period after the blood sample was taken, the majority from heart disease, cancer, and other diseases. Using statistical analysis, they looked for any links between the levels of different biomarkers in the blood and people's short-term risk of dying. They found that the levels of four biomarkers—plasma albumin, alpha-1-acid glycoprotein, very-low-density lipoprotein (VLDL) particle size, and citrate—appeared to accurately predict short-term risk of death. They repeated this study with the Finnish group, this time with 7,503 individuals (176 of whom died during the five-year follow-up period after giving a blood sample) and found similar results.

The researchers carried out further statistical analyses to take into account other known factors that might have contributed to the risk of life-threatening illness. These included factors such as age, weight, tobacco and alcohol use, cholesterol levels, and pre-existing illness, such as diabetes and cancer. The association between the four biomarkers and short-term risk of death remained the same even when controlling for these other factors.

The analysis also showed that combining the test results for all four biomarkers, to produce a biomarker score, provided a more accurate measure of risk than any of the biomarkers individually. This biomarker score also proved to be the strongest predictor of short-term risk of dying in the Estonian group. Individuals with a biomarker score in the top 20% had a risk of dying within five years that was 19 times greater than that of individuals with a score in the bottom 20% (288 versus 15 deaths).

**What Do These Findings Mean?:**

This study suggests that there are four biomarkers in the blood—alpha-1-acid glycoprotein, albumin, VLDL particle size, and citrate—that can be measured by NMR spectroscopy to assess whether otherwise healthy people are at short-term risk of dying from heart disease, cancer, and other illnesses. However, further validation of these findings is still required, and additional studies should examine the biomarker specificity and associations in settings closer to clinical practice. The combined biomarker score appears to be a more accurate predictor of risk than tests for more commonly known risk factors. Identifying individuals who are at high risk using these biomarkers might help to target preventative medical treatments to those with the greatest need.

However, there are several limitations to this study. As an observational study, it provides evidence of only a correlation between a biomarker score and ill health. It does not identify any underlying causes. Other factors, not detectable by NMR spectroscopy, might be the true cause of serious health problems and would provide a more accurate assessment of risk. Nor does this study identify what kinds of treatment might prove successful in reducing the risks. Therefore, more research is needed to determine whether testing for these biomarkers would provide any clinical benefit.

There were also some technical limitations to the study. NMR spectroscopy does not detect as many biomarkers as mass spectrometry, which might therefore identify further biomarkers for a more accurate risk assessment. In addition, because both study groups were northern European, it is not yet known whether the results would be the same in other ethnic groups or populations with different lifestyles.

In spite of these limitations, the fact that the same four biomarkers are associated with a short-term risk of death from a variety of diseases does suggest that similar underlying mechanisms are taking place. This observation points to some potentially valuable areas of research to understand precisely what's contributing to the increased risk.

**Additional Information:**

Please access these websites via the online version of this summary at http://dx.doi.org/10.1371/journal.pmed.1001606

- The US National Institute of Environmental Health Sciences has information on biomarkers
- The US Food and Drug Administration has a Biomarker Qualification Program to help researchers in identifying and evaluating new biomarkers
- Further information on the Estonian Biobank is available
- The Computational Medicine Research Team of the University of Oulu and the University of Bristol have a webpage that provides further information on high-throughput biomarker profiling by NMR spectroscopy

## Introduction

Concentrations of metabolites and proteins in the circulation can be indicative of future disease outcomes. The existing molecular biomarkers for all-cause mortality, however, display modest predictive power and risk discrimination [1],[2]. Early and accurate identification of ambulatory persons at high risk of death could assist targeting of preventive therapies. High-throughput profiling technologies for quantification of molecules from blood specimens, such as nuclear magnetic resonance (NMR) spectroscopy and mass spectrometry, have emerged as promising tools for identifying biomarkers and clarifying disease etiologies [2]–[4]. Such molecular profiling has primarily been applied to cardiometabolic diseases [3]–[5], yet a deviated circulating biomarker profile reflects systemic abnormalities and could possibly also be predictive of the risk of death from other causes [6]. Biomarkers of inflammation and hyperglycemia are associated with risk of death from cancer and other nonvascular conditions such as respiratory disease and infections, in addition to death from cardiovascular disease [7]–[9]. Novel biomarkers reflecting the risk of death from all causes hold potential to improve risk assessment, and they may further elucidate novel disease connectivities; however, high-throughput profiling of circulating biomarkers for all-cause mortality has not previously been investigated in general population settings. We therefore performed targeted screening of candidate biomarkers by NMR spectroscopy in a large, population-based study with the aim of identifying systemic biomarkers predictive of short-term risk of death from any cause. The findings were validated in an independent cohort and examined for incremental risk discrimination over and above conventional risk factors.

## Methods

### Study Populations

**Figure 1:**
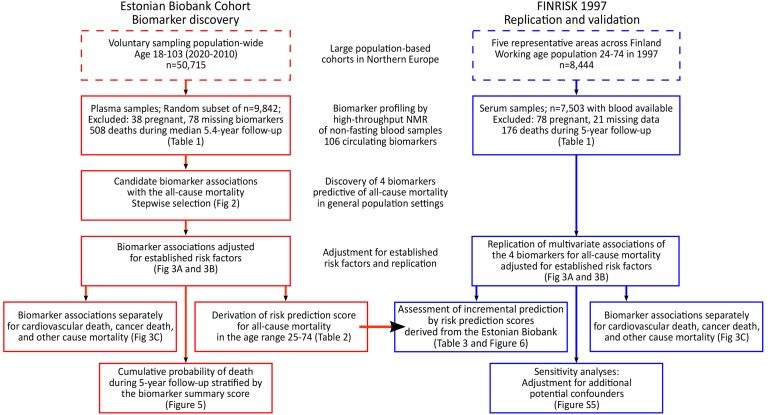
Study flow chart. Overview of the study design and analyses performed for biomarker discovery and validation of the risk prediction model.

In this observational study, two population-based cohorts in Estonia and Finland were followed for all-cause mortality via population registries. All participants provided written informed consent. The Ethics Committee of Human Studies, University of Tartu, Estonia, and the ethical committee of the National Public Health Institute, Finland, approved the studies. An overview of the study design is illustrated in Figure 1. The Estonian Biobank cohort (Estonian Genome Center, University of Tartu) included 50,715 individuals aged 18–103 y at recruitment (9 October 2002–16 February 2011), which is approximately 5% of the Estonian population within this age group. Recruitment was conducted on a voluntary basis, with no restrictions for health condition, through general practices across Estonia, as well as through recruitment centers in the two largest cities of the country [10].

Biomarker profiling was conducted by NMR spectroscopy of non-fasting plasma samples for a random subset of 9,842 individuals (pregnant women excluded). Clinical and demographic characteristics of the subset population did not differ from those of the entire cohort (*p*>0.05 for characteristics in Table 1). According to linkage with the Estonian population registry, 508 study participants had died during follow-up as of 1 June 2013.

**Table 1:** Baseline characteristics of the study participants.

Characteristic	Estonian Biobank *n* = 9,842	FINRISK 1997 *n* = 7,503
Women—number (percent)	6,334 (64%)	3,741 (50%)
Age—years (range)	45.3 (18–103)	48.4 (24–74)
Body mass index (kg/m^2^)	26.5±5.5	26.7±4.5
Systolic blood pressure (mm Hg)	126±17	136±20
Fasting duration (hours)	4.8±3.8	6.0±4.0
Total cholesterol (mmol/l)	5.4±1.1	5.5±1.1
HDL cholesterol (mmol/l)	1.7±0.4	1.4±0.4
Triglycerides (mmol/l)	1.5±1.0	1.5±1.1
Current smokers—number (percent)	2,963 (30%)	1,770 (24%)
Smoking duration (years)	8.2±13.0	10.9±13.6
Cigarettes per day	5.5±8.4	3.7±7.8
Alcohol consumption (grams/week)	29±62	25±125
Use of antihypertensive therapy—number (percent)	2,489 (25%)	1,009 (13%)
Use of lipid lowering therapy—number (percent)	413 (4.2%)	269 (0.4%)
Prevalent diabetes—number (percent)	737 (7.5%)	437 (5.8%)
Prevalent cardiovascular disease—number (percent)	899 (9.2%)	262 (3.5%)
Prevalent cancer—number (percent)	361 (3.7%)	175 (2.3%)
Alpha-1-acid glycoprotein (standardized units)	1.55±0.27	1.37±0.23
Albumin (standardized units)	101±7.5	96±6.3
VLDL particle size (average diameter, nm)	37±1.9	36±1.1
Citrate (µmol/l)	98±34	110±19

Data are mean ± SD unless otherwise indicated.

The FINRISK 1997 study is a general population study conducted to monitor the health of the Finnish population among persons aged 24–74 y at recruitment [11]. In total, 8,444 individuals were recruited to represent the working age population of five study areas across Finland [11]. Standard clinical laboratory measures were collected, and participants filled out questionnaires on physical activity and socioeconomic status. Biomarker profiling by NMR spectroscopy of serum samples was conducted for 7,503 individuals. Median fasting time was 5 h (interquartile range 4–6 h). All participants had registry-based follow-up for mortality until December 31, 2010. The coverage of the follow-up was 100% for deaths that occurred in Finland. To match the follow-up time in the discovery cohort, the analyses in the validation cohort were confined to the first 5 y of follow-up; 176 of the study participants died during this period.

### Biomarker Quantification by NMR Spectroscopy

Proton NMR spectroscopy of native plasma (Estonian Biobank cohort) and serum (FINRISK study) samples was used to quantify the concentrations of 106 circulating lipids, proteins, and metabolites. These candidate biomarkers include 85 lipoprotein lipid measures, four abundant proteins, and 17 low-molecular-weight metabolites, including amino acids, glycolysis precursors, and other small molecules (Table S1). The candidate biomarkers assayed constitute the full set of molecular measures quantified from native plasma by the targeted NMR profiling employed in this study. The high-throughput NMR platform has previously been used in various epidemiological and genetics studies [12],[13], and details of the experimental protocols, including sample preparation and spectroscopy, have been previously described [14].

### Statistical Analysis

**Figure 2:**
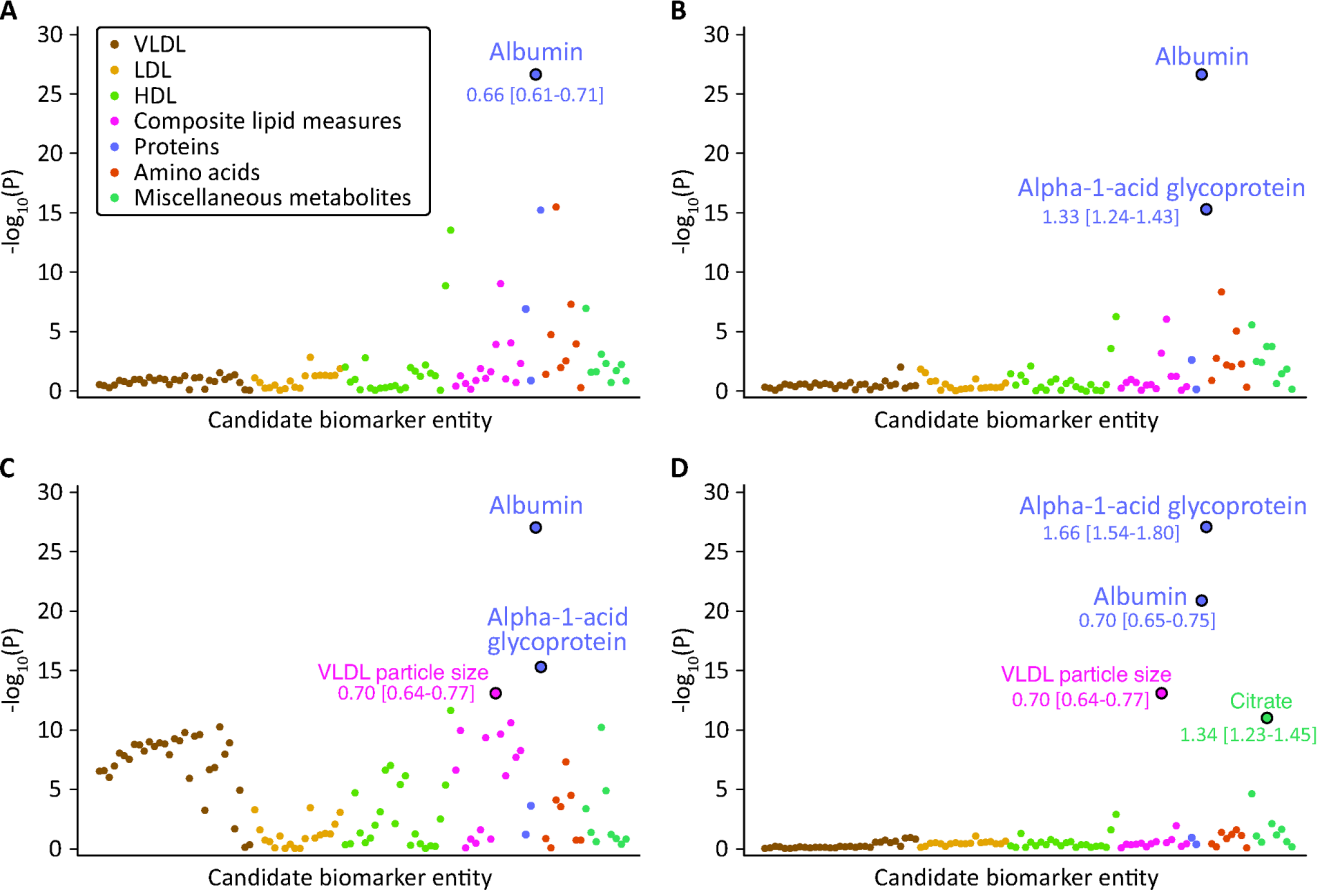
Identification of circulating biomarkers associated with the risk of all-cause mortality in the Estonian Biobank cohort. Candidate biomarkers were included in a stepwise manner into a multivariate Cox model for all-cause mortality adjusted for sex and using age as the time scale. Each biomarker is plotted against the negative log_10_ of the corresponding *p*-value. Numbers indicate HR [95% confidence interval] per 1-SD difference. Colors indicate candidate biomarker classes as listed in Table S1. (A) *p*-Values obtained when including each biomarker in turn in the model adjusted for age and sex only. Albumin was the strongest independent predictor of all-cause mortality. (B) *p*-Values for each biomarker adjusted for age, sex, and albumin. (C) *p*-Values for each biomarker adjusted for age, sex, albumin, and alpha-1-acid glycoprotein. (D) *p*-Values for each biomarker adjusted for age, sex, albumin, alpha-1-acid glycoprotein, and VLDL particle size. LDL, low-density lipoprotein.

All biomarker concentrations were scaled to standard deviation (SD) units. Cox proportional hazards models were used to assess the association of each candidate biomarker with the risk of all-cause mortality. Age at blood sampling was used as time scale—this effectively corresponds to adjusting for age [15]. For biomarker discovery in the Estonian Biobank cohort, a multivariate model was derived in a forward stepwise fashion (Figure 2). First, the biomarker leading to the smallest *p*-value in the Cox model adjusted for age and sex only was included as a predictor. Subsequently, the biomarker leading to the smallest *p*-value in the multivariate model adjusted for age, sex, and the first biomarker was included in the prediction model. The process was repeated until no additional biomarkers were significant at the Bonferroni-corrected threshold of *p*<0.0005, accounting for testing of 106 candidate biomarkers.

The hazard ratios (HRs) of the four identified biomarkers for all-cause mortality were subsequently examined in a multivariate model adjusted for age, sex, and conventional risk factors that were significant predictors of mortality in the Estonian Biobank cohort: high-density lipoprotein (HDL) cholesterol, current smoking, prevalent diabetes, prevalent cardiovascular disease, and prevalent cancer (Model A). The biomarker associations were further assessed with additional adjustment for body mass index, systolic blood pressure, total cholesterol, triglycerides, creatinine, cigarettes smoked per day, years smoked, and alcohol consumption (Model B). Proportional hazards assumptions of the regression models were confirmed by Schoenfeld's test. Sub-analyses of the four biomarkers were also conducted for cause-specific mortality. Here, analysis of cardiovascular mortality was adjusted for age, sex, blood pressure, antihypertensive treatment, current smoking, total cholesterol, HDL cholesterol, prevalent diabetes, and prevalent cardiovascular disease [16]. Analysis of cancer mortality was adjusted for age, sex, smoking, prevalent cancer, and family history of cancer. Analysis of death from nonvascular, non-cancer causes was adjusted as for Model A. Spearman's correlations between the four biomarkers and established metabolic risk factors were calculated. A biomarker summary score was derived by adding the concentrations of the biomarkers weighted by the regression coefficients (natural logarithm of HR) observed in Model A. Scatter plots of age versus the biomarker score were constructed for men and women, and the associations were examined by third degree polynomial regression fits. Kaplan-Meier plots of the 5-y cumulative mortality were calculated for quintiles and extreme quantiles of the biomarker score.

Biomarker associations with all-cause mortality in the Estonian Biobank were replicated in the FINRISK validation cohort. Cox regression models were evaluated during the first 5 y of follow-up in the FINRISK study in order to match the follow-up time in the Estonian Biobank cohort. The same set of adjustment factors was used as for the discovery cohort (see above). The incremental predictive value of the four circulating biomarkers was tested in the FINRISK validation cohort by comparing a risk prediction score composed of conventional risk factors (Model B) to a risk prediction score extended with the four biomarkers. The risk prediction scores for 5-y mortality in the FINRISK study were calculated based on the regression coefficients derived from the Estonian Biobank cohort in the age range 25–74 y (Table 2). Discrimination was assessed by C-statistics [17] and integrated discrimination improvement (IDI) accounting for censoring [18]. Net reclassification improvement (NRI) was assessed as a continuous measure [18], and by assigning participants to one of four categories (<1.25%, 1.25%–2.5%, 2.5%–5%, >5%) according to their 5-y risk of death based on the reference model and the biomarker model [19]. IDI denotes the average increase in risk estimates for persons who died during follow-up plus the average decrease in risk estimates among persons who did not die [18]. In contrast, continuous NRI indicates the percentage of individuals who died and were shifted towards higher risk plus the percentage of individuals who did not die and were shifted towards lower risk estimates, irrespective of the magnitude of altered risk [18]. Model calibration within risk deciles was assessed by the Hosmer-Lemeshow goodness-of-fit test, which compares the observed death rate with that predicted from the model. Analyses were performed with R software version 3.00 (R Foundation for Statistical Computing; http://www.r-project.org/).

**Table 2:** Hazard ratios for all-cause mortality derived in the Estonian Biobank cohort in the age range 25–74 y.

Variable	Prediction Model without Biomarkers			Prediction Model with Biomarkers		
	HR	95% CI	*p*-Value	HR	95% CI	*p*-Value
Female gender	0.67	0.50–0.90	0.009	0.60	0.44–0.81	0.0008
Body mass index^a^	1.05	0.91–1.21	0.52	1.05	0.92–1.20	0.48
Systolic blood pressure^a^	0.96	0.85–1.09	0.51	1.04	0.92–1.18	0.55
Fasting duration (hours)	0.99	0.96–1.02	0.47	1.00	0.97–1.03	0.96
Total cholesterol^a^	1.05	0.91–1.21	0.50	1.15	0.97–1.36	0.11
HDL-cholesterol^a^	0.81	0.69–0.95	0.01	1.07	0.92–1.24	0.37
Triglycerides^a^	0.82	0.70–0.96	0.01	0.93	0.71–1.21	0.60
Creatinine^a^	1.10	1.03–1.18	0.005	1.04	0.96–1.12	0.31
Current smoking	1.86	1.26–2.75	0.002	1.56	1.05–2.33	0.03
Smoking duration (years)^a^	1.21	1.04–1.41	0.01	1.25	1.07–1.46	0.005
Cigarettes per day^a^	0.93	0.80–1.07	0.29	0.89	0.77–1.03	0.11
Alcohol^a^	1.09	0.98–1.21	0.11	1.04	0.94–1.16	0.43
Prevalent diabetes	1.58	1.15–2.15	0.004	1.49	1.09–2.03	0.01
Prevalent cardiovascular disease	1.38	1.05–1.82	0.02	1.42	1.08–1.87	0.01
Prevalent cancer	2.15	1.51–3.05	2×10^−5^	2.26	1.59–3.20	5×10^−6^
Alpha-1-acid glycoprotein^a^	—	—	—	1.76	1.57–1.97	9×10^−23^
Albumin^a^	—	—	—	0.66	0.59–0.73	4×10^−15^
VLDL particle size^a^	—	—	—	0.74	0.58–0.94	0.01
Citrate^a^	—	—	—	1.47	1.29–1.67	5×10^−9^

Hazard ratios for all-cause mortality derived in the Estonian Biobank cohort in the age range matching the FINRISK cohort (25–74 y). The regression coeffients (natural logarithm of the HRs) from the Estonian Biobank cohort were used to derive two risk scores for the prediction of all-cause mortality: a reference risk score without biomarkers and a risk score including the four novel biomarkers. The two risk prediction scores were used to calculate the absolute risk estimates in the FINRISK cohort, and the incremental predictive utility of adding the four biomarkers to the risk prediction score was evaluated.

a Continuous variables were scaled to risk estimate per 1-SD increment in the variable.

## Results

The discovery analyses of biomarkers predictive of the risk for all-cause mortality comprised 9,842 individuals from the Estonian Biobank cohort with NMR-based circulating biomarker profiles. The findings were validated in a cohort of 7,503 individuals from the FINRISK study. Baseline characteristics of the study populations are shown in Table 1. During the follow-up period (median 5.4 y; range 2.4–10.7 y), there were 508 deaths among participants from the Estonian Biobank cohort: 241 deaths from cardiovascular disease, 151 from cancer, 74 from other disease-related causes, 28 from external causes, and 14 from unknown causes. In the FINRISK cohort, there were 176 deaths during 5 y of follow-up: 51 cardiovascular deaths, 68 cancer deaths, 49 deaths from other disease-related causes, and eight deaths from external causes.

The associations of the 106 candidate biomarkers with all-cause mortality are listed in Table S1. This selection of circulating metabolites and proteins represents the set of molecular measures quantified from native plasma by the high-throughput NMR profiling. Using a hypothesis-free biomarker discovery approach, four circulating biomarkers were found to be associated with all-cause mortality in a multivariate Cox model. The stepwise addition of the biomarkers to the model is illustrated in Figure 2. Plasma albumin and alpha-1-acid glycoprotein displayed strong and independent predictive associations with the risk of all-cause mortality. Once alpha-1-acid glycoprotein was included in the multivariate model, several measures of very-low-density lipoprotein (VLDL) rose in significance level, with the strongest association observed for VLDL particle size (Figure 2C). After VLDL particle size was added to the model, no additional lipoprotein measures remained significant. However, a further multivariate effect was observed for citrate: this metabolite was more strongly associated with the risk of all-cause mortality after inclusion of the three other biomarkers in the model (Figure 2D).

**Figure 3:**
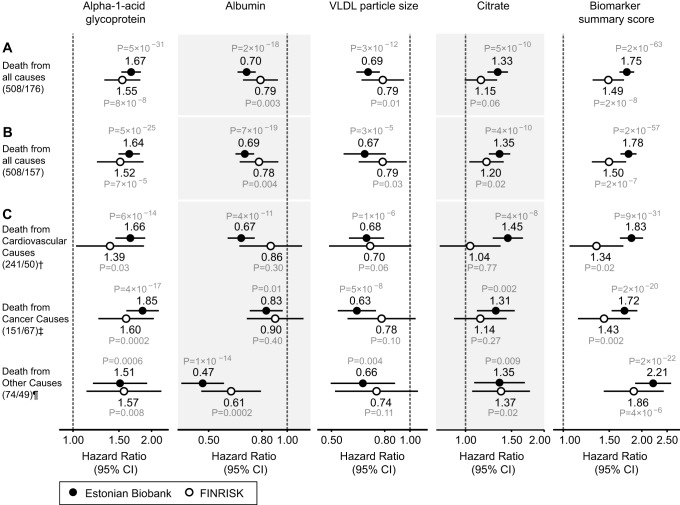
Circulating biomarkers predictive of the risk of death from all causes and cause-specific categories. (A) HRs for all-cause mortality in a multivariate Cox model adjusted for age and sex, as well as established risk factors that were significant predictors of mortality in the Estonian Biobank cohort: HDL cholesterol, current smoking, prevalent diabetes, prevalent cardiovascular disease, and prevalent cancer. HRs are per 1-SD increment in biomarker concentration. Error bars denote 95% confidence intervals. Numbers in parentheses indicate deaths during follow-up (Estonian Biobank cohort/FINRISK cohort). (B) Multivariate Cox model additionally adjusted for body mass index, systolic blood pressure, fasting time, total cholesterol, triglycerides, creatinine, smoking duration, and alcohol consumption. (C) HRs for major categories of causes of death. **†**Cardiovascular mortality was adjusted for age, sex, systolic blood pressure, current smoking, total cholesterol, HDL cholesterol, antihypertensive treatment, prevalent cardiovascular disease, and prevalent diabetes. **‡**Cancer mortality was adjusted for age, sex, smoking status, prevalent cancer, and family history of cancer. **¶**Other disease-related mortality was adjusted as for (A).

The four circulating biomarkers were associated with all-cause mortality to a similar extent when adjusted for conventional risk factors that were significant predictors of mortality in the Estonian Biobank cohort (HDL cholesterol, current smoking, and prevalent disease): alpha-1-acid glycoprotein (adjusted HR 1.67 per 1-SD concentration increment, 95% CI 1.53–1.82), albumin (HR 0.70, 95% CI 0.65–0.76), VLDL particle size (HR 0.69, 95% CI 0.62–0.77), and citrate (HR 1.33, 95% CI 1.21–1.45). All four biomarkers were also associated with all-cause mortality during 5 y of follow-up in the FINRISK validation cohort, with consistent HRs (Figure 3A). The results were essentially unaltered when further adjusting for additional confounders including body mass index, blood pressure, lipids, and creatinine (Figure 3B). The four biomarkers were further found to be predictive of the risk of death across three major categories of deaths in the Estonian Biobank cohort: cardiovascular deaths, cancer deaths, and deaths from other disease-related causes (Figure 3C). For most of the biomarker associations, the HR estimates for cause-specific mortality were concordant, albeit weaker, in the FINRISK cohort. Correlations between the four biomarkers and established metabolic risk factors are shown in Figure S1. Notably, elevated VLDL particle size was associated with decreased risk of death (Figure 3), despite the fact that the measure is strongly positively correlated with alpha-1-acid glycoprotein (*r* = 0.53) and triglyceride levels (*r* = 0.82). The multivariate effect observed for alpha-1-acid glycoprotein and VLDL particle size, with the two biomarkers being more strongly associated with the risk of death when both measures were included in the model, is further illustrated in Figure S2. Moreover, when the four circulating biomarkers were included in the model, the measures of total and HDL cholesterol, as well as triglycerides, were not significant predictors of all-cause mortality (Table 2).

**Figure 4:**
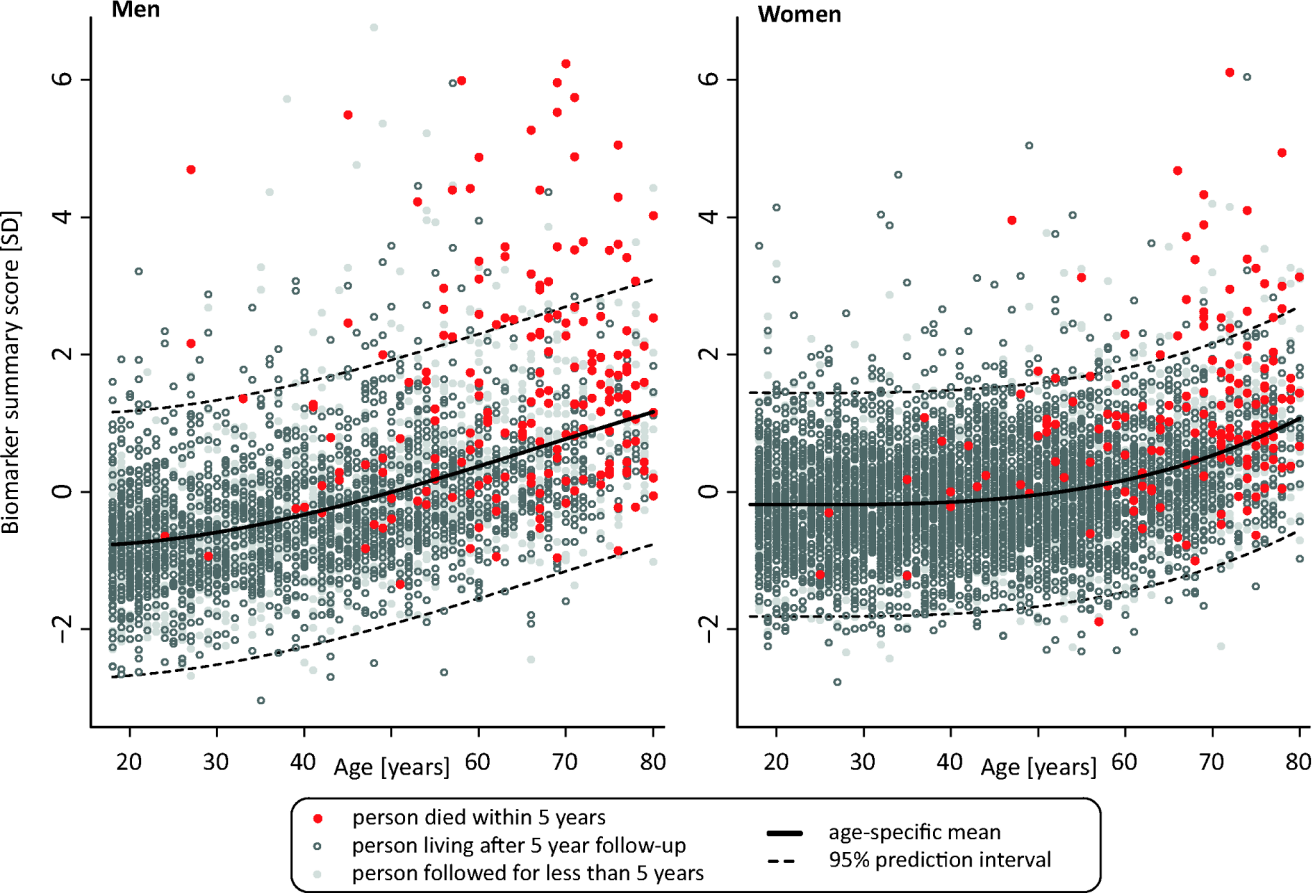
Scatter plot of age versus biomarker summary score for men and women from the Estonian Biobank cohort. The lines indicate a fit of age against the biomarker summary score, with dashed lines denoting 95% prediction intervals. Persons who died within the 5-y follow-up period are marked by red dots, and persons who were alive after 5 y are marked by open gray circles. Persons with less than 5 y of follow-up are marked in light gray.

**Figure 5:**
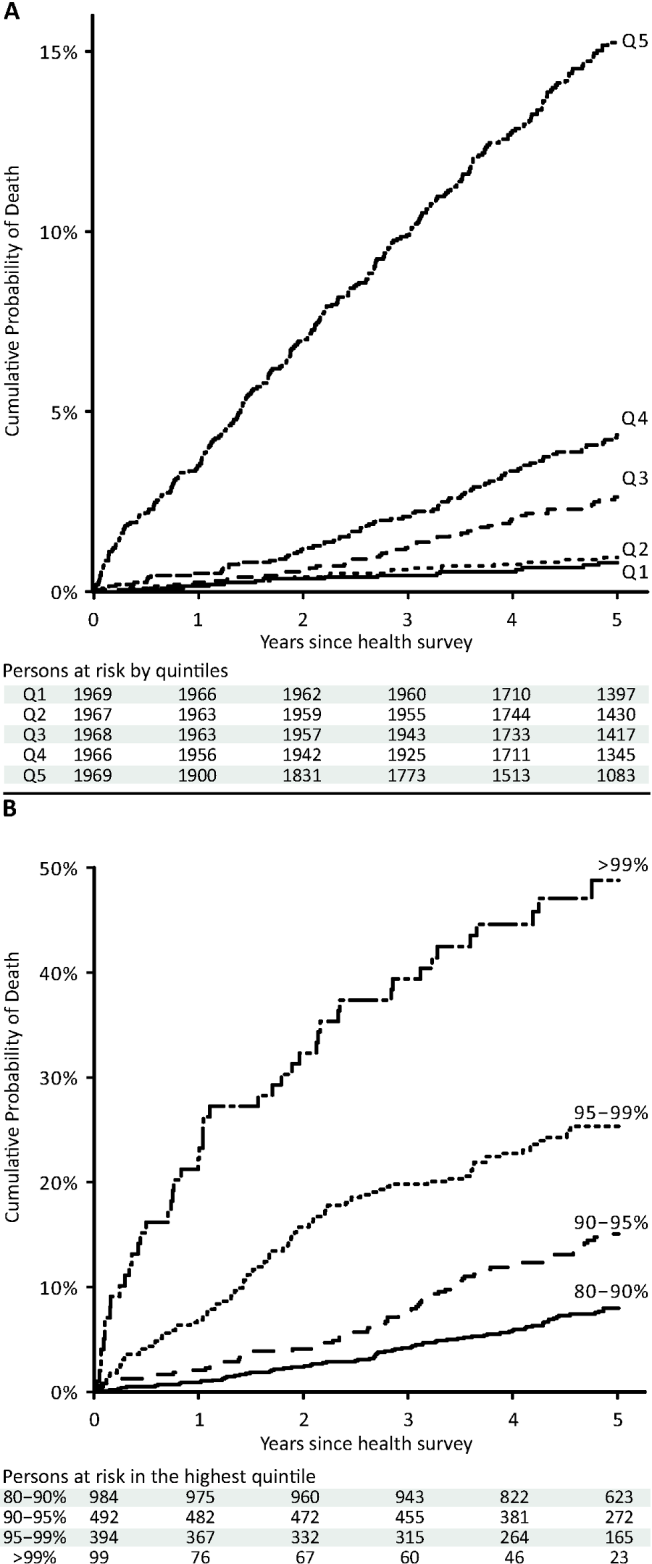
Cumulative probability of death in the Estonian Biobank cohort by percentiles of the biomarker summary score. The 5-y cumulative mortality is shown per quintile of the biomarker summary score (A) and with further stratification of the highest quintile (B).

A biomarker summary score was calculated as the sum of the four biomarker concentrations weighted by the regression coefficients. The biomarker score was the strongest predictor of short-term risk of death among all risk factors available in the Estonian Biobank cohort. The association of the biomarker score with age is illustrated in Figure 4. The biomarker score was moderately correlated with age (*r* = 0.38), yet extreme biomarker score values were seen across all age groups. Excess mortality within 5 y of follow-up was observed for higher age, but in particular in combination with an elevated biomarker score (Figure 4); however, the association of the biomarker score with all-cause mortality was generally similar across age groups (*p* = 0.48 for interaction with age). To illustrate the strong association of the biomarker summary score in the Estonian Biobank cohort, the cumulative probability of death was derived across quintiles of the biomarker score (Figure 5A). The 5-y mortality for persons with a biomarker score within the highest quintile was 19 times higher than for those in the lowest quintile (288 versus 15 deaths during 5 y, corresponding to 15.3% versus 0.8%). Individuals within the highest quintile were further differentiated in terms of their short-term probability of dying according to their biomarker score percentiles: 23% of the individuals with a biomarker score within the highest percentile had died within the first year of follow-up (23 out of 99), and the estimated 5-y mortality was 49% (Figure 5B).

### Risk Score Validation and Risk Discrimination

**Figure 6:**
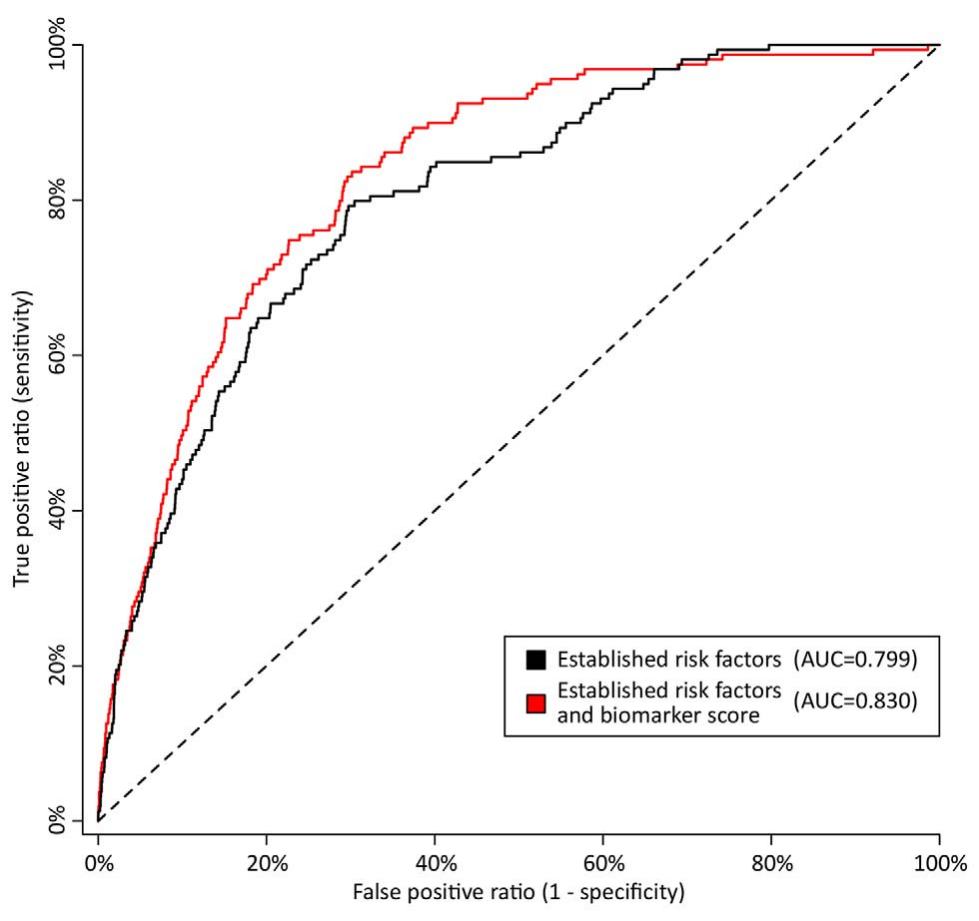
Discrimination curves for 5-y mortality in FINRISK cohort. Receiver operating characteristic curves from risk prediction scores based on conventional risk factors (black) and with the biomarkers alpha-1-acid glycoprotein, albumin, VLDL particle size, and citrate included in the risk prediction score (red). The risk assessment was evaluated in the FINRISK cohort based on risk scores derived from the Estonian Biobank cohort. Conventional risk factors are age, sex, body mass index, systolic blood pressure, fasting time, total cholesterol, HDL cholesterol, triglycerides, creatinine, smoking, alcohol, prevalent diabetes, prevalent cardiovascular disease, and prevalent cancer. AUC, area under the curve.

To illustrate the potential of the circulating biomarkers to improve risk discrimination for all-cause mortality in an independent cohort, risk prediction scores for all-cause mortality with and without the biomarkers were derived in the Estonian Biobank cohort and evaluated in the FINRISK validation cohort. The regression coefficients used for calculating the two risk scores are listed in Table 2. A risk prediction score for 5-y mortality composed of conventional risk factors was compared to a risk score extended with the four circulating biomarkers (Table 3). Risk discrimination was significantly improved by including the biomarkers in the risk prediction score in terms of the C-statistics (0.031 increase, *p* = 0.01) and the IDI (1.9%, *p* = 0.02). The discrimination curves are illustrated in Figure 6. For reclassication, a continuous NRI of 26.3% (*p* = 0.001) was achieved when incorporating the four biomarkers into the risk prediction score. Specifically, 81 out of the 157 persons who died during the 5-y follow-up were shifted towards higher risk estimates, while 76 were shifted downwards in risk (net 3.1%); among the 6,953 individuals who did not die, 4,283 persons were shifted towards lower risk estimates and 2,670 were shifted upwards in risk (net 23.2%). The category-based NRI was 9.2% (*p* = 0.08) when persons were assigned to one of four groups (<1.25%, 1.25%–2.5%, 2.5%–5%, >5%) according to their 5-y risk of death. The category-based reclassification was driven by down-classification of risk among persons who did not die during the 5-y follow-up (7.9%, *p* = 2×10^−24^), as detailed in Table S2. Model calibration was adequate for both risk scores when the numbers of deaths observed within risk deciles were compared with the death rates predicted from the models (*p*>0.01, Figure S3).

**Table 3:** Discrimination and reclassification for 5-y all-cause mortality in the FINRISK cohort with and without circulating biomarkers in the risk prediction score.

Reference Risk Score C-Statistic	Biomarker Risk Score C-Statistic	Difference in C-Statistic	IDI	Continuous NRI	Category-Based NRI
0.799	0.830	0.031±0.012 *p* = 0.01	1.9±0.8% *p* = 0.02	26.3±8.0% *p* = 0.001	9.2±5.4% *p* = 0.08

Risk discrimination was assessed for FINRISK study participants with the risk prediction scores derived in the Estonian Biobank cohort (Table 2). The reference risk score included age, sex, body mass index, systolic blood pressure, fasting time, total cholesterol, HDL cholesterol, triglycerides, creatinine, smoking status, alcohol consumption, prevalent diabetes, prevalent cardiovascular disease, and prevalent cancer. The biomarker risk prediction score was extended with alpha-1-acid glycoprotein, albumin, VLDL particle size, and citrate. Complete data were available for 7,110 individuals, of which 157 died during the 5-y follow-up period. Category-based reclassification was assessed for four risk categories (≤1.25%, 1.25%–2.5%, 2.5%–5%, ≥5%) based on the reference risk score and the biomarker risk score. Reclassification tables for these groups and model calibration of the prediction scores within risk deciles are shown in Figure S3 and Table S2.

### Sensitivity Analyses

The biomarker associations were consistent for both men and women (Table S3); there was no significant modulation of hazard when sex interaction terms with all four biomarkers were added to the model (*p*>0.05). To examine the biomarker associations with all-cause mortality among apparently healthy persons, we conducted analyses excluding persons with prevalent diabetes, cardiovascular disease, and cancer in both cohorts. Here, all four circulating biomarkers remained predictive of the risk of death with essentially unaltered HRs (Figure S4). The better match of the biomarker associations between the two cohorts among persons free of apparent disease suggests that the minor discrepancies of the HRs observed in Figure 3 can partly be attributed to differences in prevalent disease. In the FINRISK study, information was available on household income, leisure time physical activity index, and C-reactive protein; all biomarker associations were broadly similar when these potential confounders were included in the model (Figure S5). Adjusting for or excluding individuals on lipid-lowering or antihypertensive treatment from analyses did not change the findings (Figure S5). Results were also similar when individuals who died within the first year of follow-up were excluded (Figure S5).

## Discussion

Four circulating biomarkers—alpha-1-acid glycoprotein, albumin, VLDL particle size, and citrate—were predictive of the short-term risk of death from any cause in two general population cohorts. All four biomarkers were not only associated with cardiovascular mortality, but were also indicators of the risk of cancer death and other nonvascular causes of mortality. In combination, the biomarkers improved risk discrimination and reclassification over and above conventional risk factors and may potentially aid the identification of high-risk individuals in need of medical intervention. Although the clinical implications remain unclear in terms of disease specificity and treatment strategies, these findings illustrate the utility of population-level molecular profiling for biomarker discovery, and suggest systemic reflections of the risk for death across disparate disease causes [7],[20].

The four biomarkers associated with all-cause mortality among ambulatory people are implicated in various pathophysiological mechanisms including inflammation, fluid imbalance, lipoprotein metabolism, and metabolic homeostasis. The acute phase protein alpha-1-acid glycoprotein (also known as orosomucoid) is elevated in response to infection and inflammation [21]. Plasma levels of alpha-1-acid glycoprotein have been associated with all-cause mortality in elderly persons, as well as cardiovascular mortality and prognosis of certain cancers [22]–[24]. Here, alpha-1-acid glycoprotein was the strongest multivariate predictor of the risk of death from all causes. Once added to the prediction model, alpha-1-acid glycoprotein additionally influenced the association of several VLDL lipid measures with all-cause mortality (Figure 2). The association of alpha-1-acid glycoprotein with mortality was only slightly attenuated when C-reactive protein, a widely used marker of low-grade inflammation, was included in the prediction model (Figure S5). The functional role of alpha-1-acid glycoprotein remains poorly understood; however, these findings support the notion of acute phase proteins being reflective of the risk of death from vascular and nonvascular disease, as well as cancer [7].

Plasma albumin, as available from a routine blood test, is a marker of liver and kidney function, nutritional status, and inflammation [25]. Low circulating albumin levels are associated with increased mortality from vascular, nonvascular, and cancer causes, both in apparently healthy persons and acutely ill patients [7],[25],[26]. The strong inverse association of albumin with short-term risk of death may therefore be considered as a positive control in the biomarker discovery. Although hypoalbuminemia has been linked with susceptibility to various diseases and can be used as a marker of frailty in older people [27], the general population variation in albumin levels is not routinely used for risk assessment among asymptomatic persons.

Triglyceride-mediated lipoprotein metabolism is recognized as a risk factor for cardiovascular disease, particularly in the non-fasting state [28],[29]. VLDL particles are the starting point of the hepatic lipoprotein cascade, and the average size of VLDL particles may be an overall indicator of triglyceride metabolism. In this study, VLDL particle size was inversely associated with risk of death, and the association became stronger when alpha-1-acid glycoprotein was included in the multivariate model (Figures 2C and S2). This might indicate a combined effect of perturbed triglyceride metabolism and low-grade inflammation, as has been supported by genetic evidence [30]. Although postprandial triglyceride levels have been linked with all-cause mortality [29], measures of VLDL and triglyceride metabolism have not previously been associated with cancer mortality or death from other nonvascular causes.

Citrate is an intermediate in the Krebs cycle and thus central to energy metabolism. Circulating citrate levels are tightly regulated, since citrate acts as a chelator to modulate calcium, magnesium, and zinc ion concentrations, and thereby exhibits anticoagulating properties [31]. However, citrate has not been previously implicated as a biomarker for mortality in general population settings. Increased citrate was associated with increased risk of cardiovascular death as well as cancer death among participants in the Estonian Biobank cohort; however, the most consistent associations were observed for deaths from other causes (Figure 3C). A recent molecular profiling study indicated citric acid cycle deviations, including elevated citrate levels, as being predictive of death from sepsis in hospital settings [9]. The mechanisms underlying how citrate is associated with short-term risk of death among ambulatory people nonetheless remain elusive.

Out of all available risk factors, the biomarker summary score was the strongest predictor of all-cause mortality in the Estonian Biobank cohort. The biomarker score stratified the short-term risk of death: persons with a very high biomarker score were associated with substantially higher mortality rates than those with average levels of the biomarker score, indicating prominent reflections of frailty in the systemic biomarker profile (Figure 5). Importantly, all hazard estimates were similar in analyses limited to individuals without prevalent diabetes, cardiovascular disease, or cancer (Figure S4). If these findings are further validated, it might be envisioned that NMR-based biomarker profiling of non-fasting blood specimens could be helpful for identifying asymptomatic people at high risk to be referred for more detailed screening procedures. Additional studies are, however, still required to elucidate the disease specificity and etiological underpinnings of the biomarker associations, as well as inform potential treatment strategies. For these reasons, the risk prediction model for all-cause mortality (Tables 2 and 3) should serve only as an illustration of the potential to enhance risk discrimination; evaluation of the predictive utility of the biomarkers in settings closer to clinical practice are called for to clarify implications for public health intervention.

Although the associations of the four biomarkers were largely unaffected by potential confounders (Figures 3 and S5), it is still plausible that subclinical or overt disease processes may underpin the biomarker reflections of the short-term risk of death. Co-morbidities such as respiratory, renal, and liver disease could partly mediate the biomarker associations; additional studies are warranted to address the effects of low-grade inflammation, infection, and prevalent disease on the biomarker concentrations. Importantly, the strong associations do not imply causal influences of the biomarkers on the risk of death. Notwithstanding, the biomarker associations across cardiovascular, nonvascular, and cancer mortality open a host of pathophysiological questions, and highlight latent systemic connectivities across seemingly dissimilar causes of death.

Some limitations of our study should be considered. The molecular coverage available from NMR spectroscopy is limited compared to that afforded by mass spectrometry, which holds further promise for risk assessment and elucidation of disease pathways [2],[32]. Both plasma and serum samples were non-fasting, and generalization to fasting biomarker concentrations requires further studies. Albumin and lipoprotein levels are, however, only weakly associated with fasting duration [33]; all results were similar when adjusting for time since last meal. The risk of all-cause mortality is not customarily assessed in general practice, and no established risk categories exist to guide treatment; nonetheless, progress towards enhanced risk prediction accuracy may enable applications for targeted prevention. This study was conducted in two independent cohorts of northern European individuals; further evaluation of the biomarkers in other lifestyle environments and ethnic groups is warranted.

In summary, high-throughput molecular profiling by NMR spectroscopy highlighted four circulating biomarkers—alpha-1-acid glycoprotein, albumin, VLDL particle size, and citrate—predictive of the short-term risk of death from all causes. The biomarker associations were replicated in an independent population and were consistent when limiting analyses to persons free of apparent disease. All four biomarkers were predictive of death from cancer and nonvascular causes in addition to cardiovascular mortality, and may therefore indicate novel relationships between systemic biomarkers and diverse morbidities. Incorporating the biomarkers into risk prediction scores led to improved discrimination and reclassification of 5-y mortality in the validation cohort. Further investigations are required to clarify the utility of these circulating biomarkers for guiding screening and targeted prevention based on the molecular profile of an individual.

## Supporting Information

Figure S1 **Correlations between biomarkers for mortality and metabolic risk factors.**
(PDF)

Figure S2 **Scatter plot of very-low-density lipoprotein particle size versus alpha-1-acid glycoprotein and observed mortality in the Estonian Biobank cohort.**
(PDF)

Figure S3 **Calibration of risk prediction scores for 5-y all-cause mortality in the FINRISK cohort.**
(PDF)

Figure S4 **Hazard ratios for all-cause mortality among individuals free of apparent disease at baseline.**
(PDF)

Figure S5 **Hazard ratios for all-cause mortality upon adjustment for potential confounders in the FINRISK cohort.**
(PDF)

Table S1 **Circulating candidate biomarkers quantified by high-throughput NMR profiling.**
(PDF)

Table S2 **Reclassification tables of individuals who died and who did not die during 5 y of follow up in the FINRISK cohort.**
(PDF)

Table S3 **Hazard ratios for all-cause mortality in the Estonian Biobank cohort stratified by gender.**
(PDF)

## Abbreviations

HDL: high-density lipoprotein
HR: hazard ratio
IDI: integrated discrimination improvement
NMR: nuclear magnetic resonance
NRI: net reclassification improvement
SD: standard deviation
VLDL: very-low-density lipoprotein
