# Supplementary material for: Biomarker Profiling by Nuclear Magnetic Resonance Spectroscopy for the Prediction of All-Cause Mortality: An Observational Study of 17,345 Persons
Source: PLoS Med. 2014 Feb 25;11(2):e1001606. doi: 10.1371/journal.pmed.1001606 (PMC3934819; doi:10.1371/journal.pmed.1001606)
Supplement: Figure S1 — Correlations between biomarkers for mortality and metabolic risk factors. (PDF) [file pmed.1001606.s001.pdf]

Figure S1. Correlations between biomarkers for mortality and metabolic risk factors.

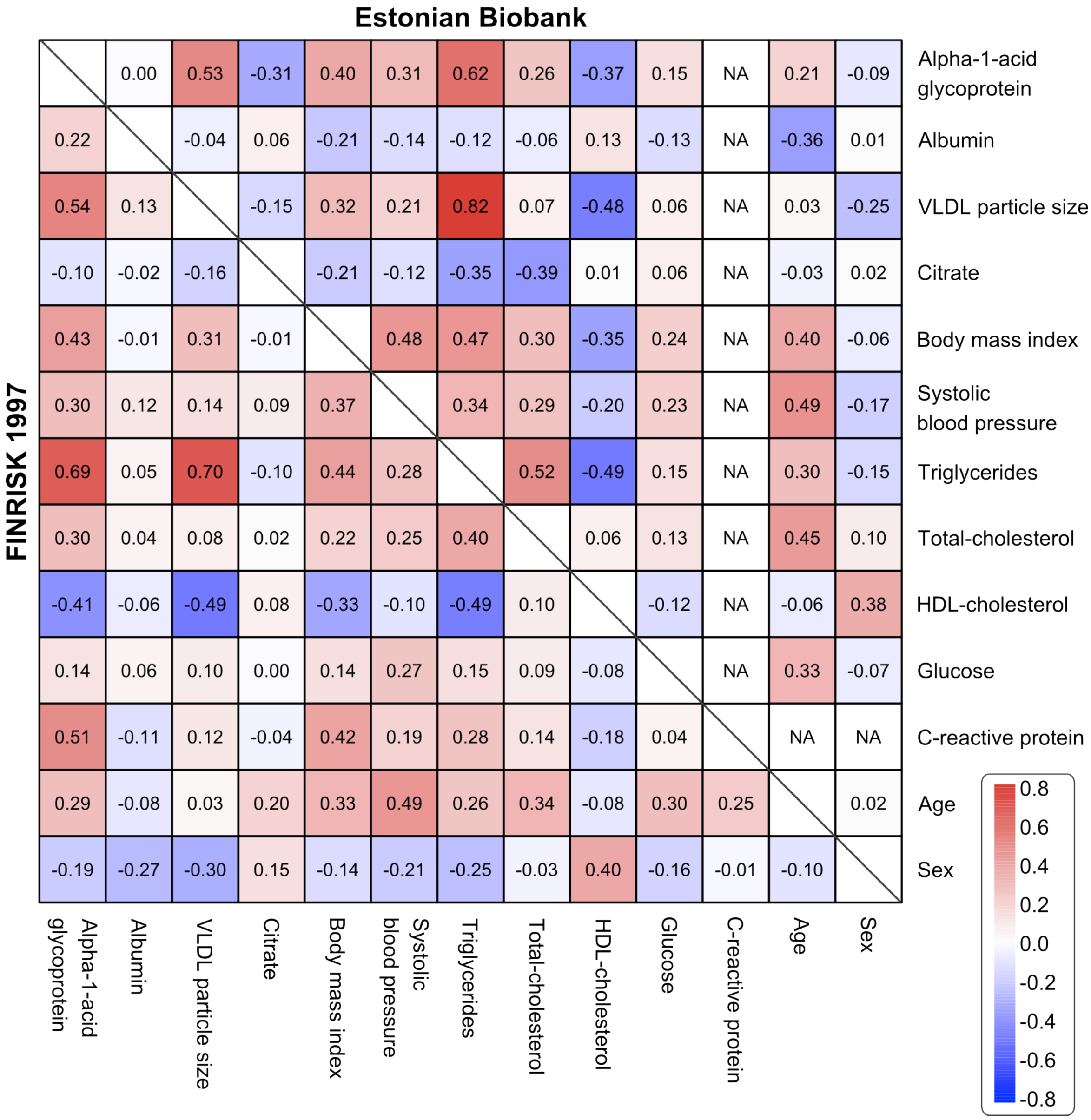

Correlation map of the four circulating biomarkers associated with all-cause mortality and established metabolic risk factors. Numbers indicate Spearman's correlation coefficients in the Estonian Biobank cohort (upper triangle) and the FINRISK 1997 cohort (lower triangle).
