# Supplementary material for: Biomarker Profiling by Nuclear Magnetic Resonance Spectroscopy for the Prediction of All-Cause Mortality: An Observational Study of 17,345 Persons
Source: PLoS Med. 2014 Feb 25;11(2):e1001606. doi: 10.1371/journal.pmed.1001606 (PMC3934819; doi:10.1371/journal.pmed.1001606)
Supplement: Figure S2 — Scatter plot of very-low-density lipoprotein particle size versus alpha-1-acid glycoprotein and observed mortality in the Estonian Biobank cohort. (PDF) [file pmed.1001606.s002.pdf]

Figure S2. Scatter plot of very-low-density lipoprotein particle size vs. alpha-1-acid glycoprotein and observed mortality in the Estonian Biobank cohort.

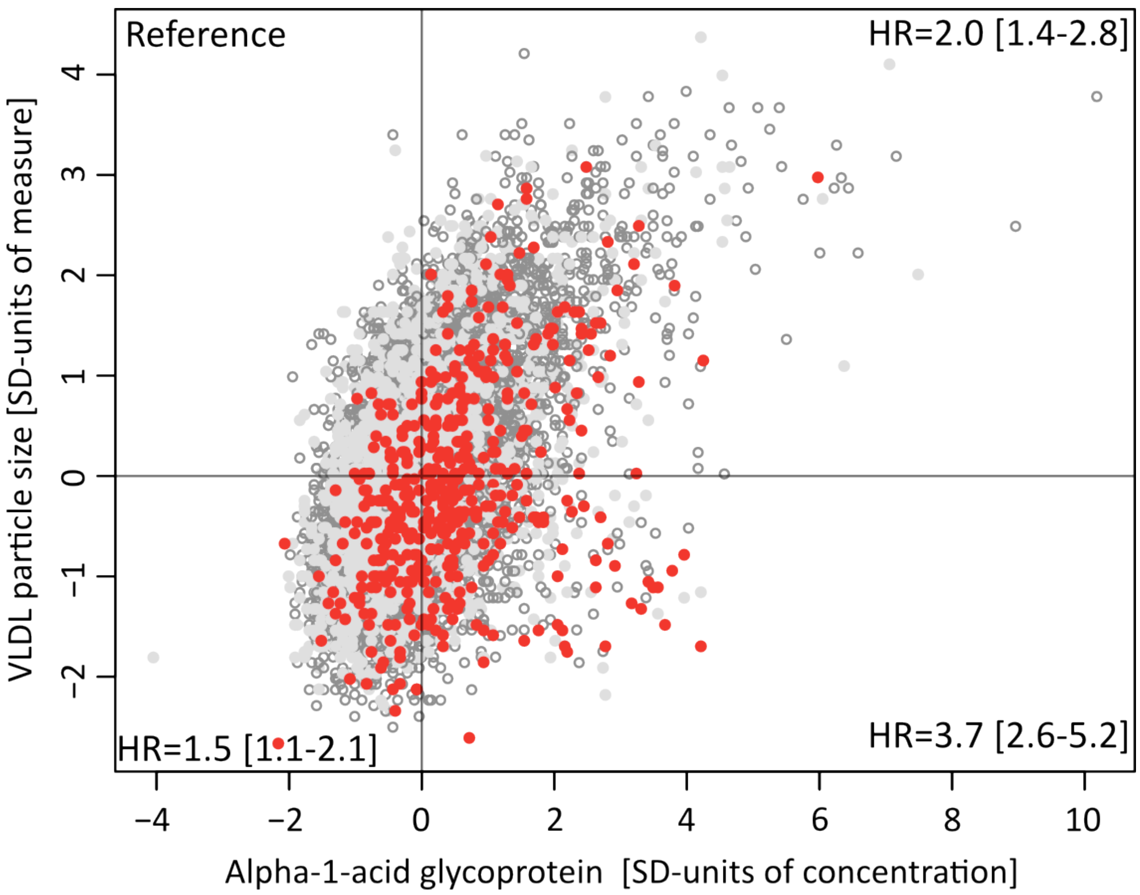

Illustration of the oppositely directed associations with mortality for alpha-1-acid glycoprotein and VLDL particle size. Both biomarkers measures are standardized to zero mean and shown in SD-units, so that the concentrations below or above the average are separated by the axes. Numbers denote the hazard ratio [95% confidence intervals] of all-cause mortality in each quadrant, compared to the low alpha-1-acid glycoprotein and large VLDL particle size reference quadrant. Despite the positive correlation (r=0.53), the two biomarkers display different direction of effect with mortality, leading to a multivariate association. Red dots indicate persons who died within 5-year follow up, and open gray circles mark persons who were alive after 5 years. Persons with less than 5 years of follow-up are indicated in light gray.
