# Supplementary material for: Biomarker Profiling by Nuclear Magnetic Resonance Spectroscopy for the Prediction of All-Cause Mortality: An Observational Study of 17,345 Persons
Source: PLoS Med. 2014 Feb 25;11(2):e1001606. doi: 10.1371/journal.pmed.1001606 (PMC3934819; doi:10.1371/journal.pmed.1001606)
Supplement: Figure S3 — Calibration of risk prediction scores for 5-y all-cause mortality in the FINRISK cohort. (PDF) [file pmed.1001606.s003.pdf]

Figure S3. Calibration of risk prediction scores for 5-year all-cause mortality in the FINRISK cohort.

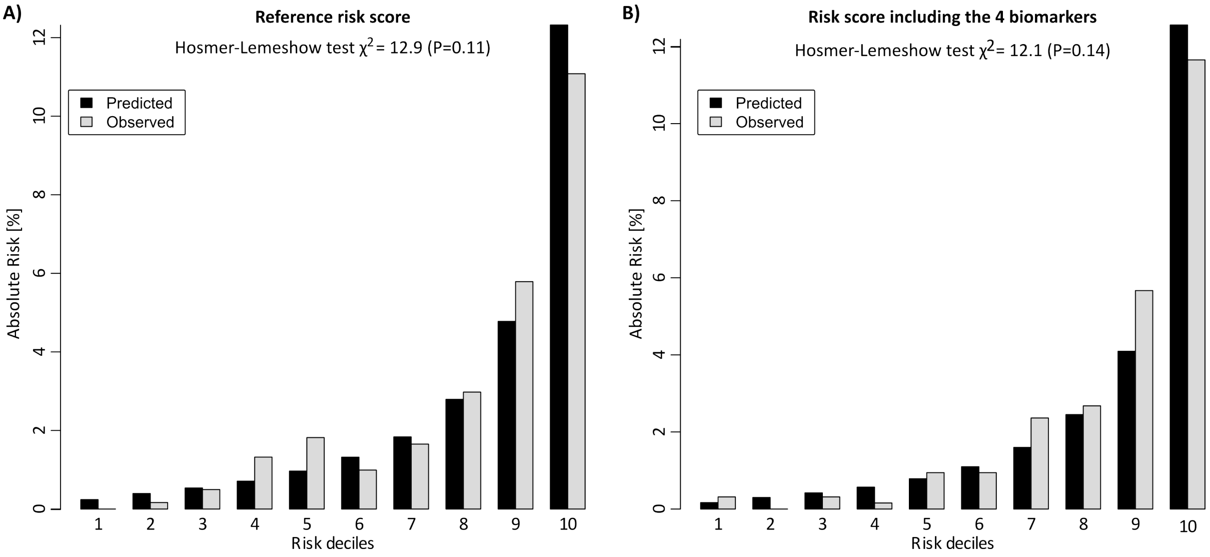

Calibration of the reference risk prediction score composed of conventional risk factors (Panel A), and the corresponding calibration when the four circulating biomarkers were included in the risk prediction score (Panel B). The absolute risk of death during 5-year follow-up was calculated in the FINRISK study based on the risk scores derived in the Estonian Biobank cohort. Calibration was examined using the Hosmer-Lemeshow goodness-of-fit test, which compares the observed number of death with those predicted from the risk score.
