# Supplementary material for: Biomarker Profiling by Nuclear Magnetic Resonance Spectroscopy for the Prediction of All-Cause Mortality: An Observational Study of 17,345 Persons
Source: PLoS Med. 2014 Feb 25;11(2):e1001606. doi: 10.1371/journal.pmed.1001606 (PMC3934819; doi:10.1371/journal.pmed.1001606)
Supplement: Figure S4 — Hazard ratios for all-cause mortality among individuals free of apparent disease at baseline. (PDF) [file pmed.1001606.s004.pdf]

Figure S4. Hazard ratios for all-cause mortality among individuals free of apparent disease at baseline.

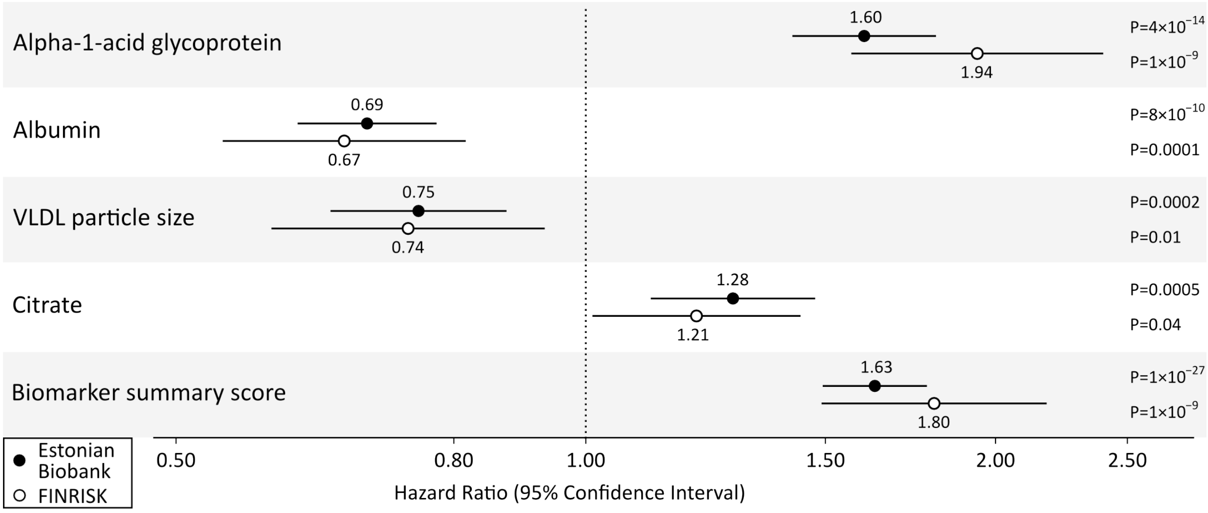

Hazard ratios (95% confidence interval) for all-cause mortality among individuals free of prevalent diabetes, cardiovascular disease, or cancer in the Estonian Biobank cohort (●; 178 deaths among 8,154 individuals) and the FINRISK cohort (○; 112 deaths among 6,557 individuals). Hazard ratios are per 1-SD increment in biomarker concentration. The multivariate Cox model was adjusted as for FIGURE 3A: age, sex, HDL cholesterol, and current smoking.
