# Supplementary material for: Biomarker Profiling by Nuclear Magnetic Resonance Spectroscopy for the Prediction of All-Cause Mortality: An Observational Study of 17,345 Persons
Source: PLoS Med. 2014 Feb 25;11(2):e1001606. doi: 10.1371/journal.pmed.1001606 (PMC3934819; doi:10.1371/journal.pmed.1001606)
Supplement: Figure S5 — Hazard ratios for all-cause mortality upon adjustment for potential confounders in the FINRISK cohort. (PDF) [file pmed.1001606.s005.pdf]

Figure S5. Hazard ratios for all-cause mortality after adjustment for additional potential confounders in the FINRISK cohort.

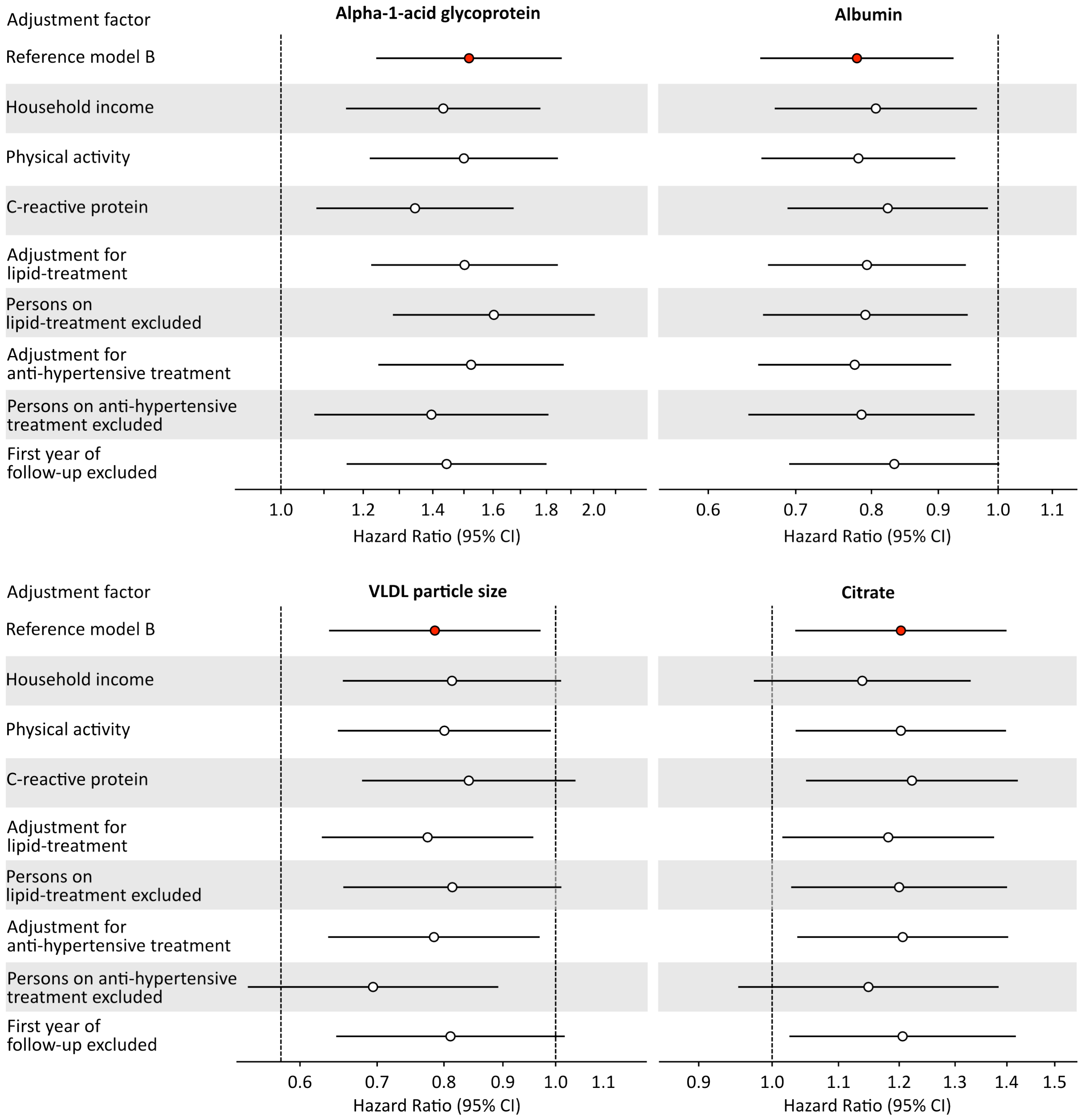

Hazard ratios for all-cause mortality per 1-SD biomarker concentration with additional adjustment for environmental factors, potential confounders and medication status in the FINRISK cohort. All models were adjusted for age, sex, body mass index, systolic blood pressure, fasting time, total cholesterol, HDL cholesterol, triglycerides, creatinine, smoking, alcohol consumption, prevalent diabetes, prevalent cardiovascular disease, and prevalent cancer. Error bars indicate 95% confidence intervals. Household income, leisure time physical activity index and medication status were assessed from questionnaires.
