# Supplementary material for: Biomarker Profiling by Nuclear Magnetic Resonance Spectroscopy for the Prediction of All-Cause Mortality: An Observational Study of 17,345 Persons
Source: PLoS Med. 2014 Feb 25;11(2):e1001606. doi: 10.1371/journal.pmed.1001606 (PMC3934819; doi:10.1371/journal.pmed.1001606)
Supplement: Table S1 — Circulating candidate biomarkers quantified by high-throughput NMR profiling. (PDF) [file pmed.1001606.s006.pdf]

Table S1. Circulating candidate biomarkers quantified by high-throughput NMR profiling.

Lipoprotein lipids	Mean±SD	Hazard ratio for mortality*
Phospholipids in chylomicrons and extremely large VLDL (mmol/L)	0.0038±0.0057	0.95 [0.87–1.04]; P=0.27
Total lipids in chylomicrons and extremely large VLDL (mmol/L)	0.031±0.047	0.96 [0.88–1.05]; P=0.35
Concentration of chylomicrons and extremely large VLDL particles (nmol/L)	0.090±0.16	0.97 [0.89–1.06]; P=0.49
Phospholipids in very large VLDL (mmol/L)	0.017±0.023	0.95 [0.87–1.04]; P=0.31
Triglycerides in very large VLDL (mmol/L)	0.068±0.091	0.93 [0.85–1.02]; P=0.12
Total lipids in very large VLDL (mmol/L)	0.10±0.13	0.94 [0.85–1.03]; P=0.15
Concentration of very large VLDL particles (nmol/L)	0.96±1.3	0.95 [0.86–1.03]; P=0.22
Total cholesterol in large VLDL (mmol/L)	0.087±0.095	0.93 [0.85–1.01]; P=0.10
Free cholesterol in large VLDL (mmol/L)	0.047±0.052	0.93 [0.85–1.01]; P=0.10
Phospholipids in large VLDL (mmol/L)	0.075±0.085	0.95 [0.86–1.03]; P=0.21
Triglycerides in large VLDL (mmol/L)	0.24±0.29	0.92 [0.84–1.01]; P=0.07
Cholesterol esters in large VLDL (mmol/L)	0.041±0.044	0.93 [0.85–1.02]; P=0.12
Total lipids in large VLDL (mmol/L)	0.41±0.48	0.92 [0.84–1.01]; P=0.09
Concentration of large VLDL particles (nmol/L)	6.7±7.9	0.93 [0.85–1.01]; P=0.10
Total cholesterol in medium VLDL (mmol/L)	0.22±0.14	0.95 [0.87–1.04]; P=0.27
Free cholesterol in medium VLDL (mmol/L)	0.098±0.075	0.93 [0.85–1.01]; P=0.10
Phospholipids in medium VLDL (mmol/L)	0.15±0.11	0.93 [0.86–1.02]; P=0.13
Triglycerides in medium VLDL (mmol/L)	0.40±0.37	0.91 [0.84–1.00]; P=0.05
Cholesterol esters in medium VLDL (mmol/L)	0.12±0.07	0.99 [0.91–1.07]; P=0.74
Total lipids in medium VLDL (mmol/L)	0.77±0.63	0.92 [0.85–1.01]; P=0.08
Concentration of medium VLDL particles (nmol/L)	21±18	0.92 [0.84–1.01]; P=0.07
Total cholesterol in small VLDL (mmol/L)	0.33±0.11	0.99 [0.91–1.07]; P=0.72
Free cholesterol in small VLDL (mmol/L)	0.13±0.047	0.94 [0.86–1.02]; P=0.12
Phospholipids in small VLDL (mmol/L)	0.19±0.07	0.94 [0.87–1.02]; P=0.16
Triglycerides in small VLDL (mmol/L)	0.30±0.19	0.91 [0.83–0.99]; P=0.03
Total lipids in small VLDL (mmol/L)	0.82±0.36	0.93 [0.86–1.02]; P=0.11
Concentration of small VLDL particles (nmol/L)	37±18	0.92 [0.85–1.00]; P=0.06
Phospholipids in very small VLDL (mmol/L)	0.17±0.059	1.09 [1.00–1.18]; P=0.04
Triglycerides in very small VLDL (mmol/L)	0.13±0.056	0.95 [0.87–1.03]; P=0.18
Total lipids in very small VLDL (mmol/L)	0.58±0.18	1.01 [0.93–1.10]; P=0.78
Concentration of very small VLDL particles (nmol/L)	39±13	1.01 [0.93–1.09]; P=0.81
Free cholesterol in IDL (mmol/L)	0.22±0.075	1.07 [0.99–1.16]; P=0.08
Phospholipids in IDL (mmol/L)	0.31±0.1	1.06 [0.97–1.14]; P=0.19
Total lipids in IDL (mmol/L)	1.2±0.37	1.02 [0.94–1.11]; P=0.58
Concentration of IDL particles (nmol/L)	97±31	1.03 [0.95–1.11]; P=0.53
Total cholesterol in large LDL (mmol/L)	1.0±0.33	0.96 [0.88–1.04]; P=0.30
Free cholesterol in large LDL (mmol/L)	0.27±0.083	1.01 [0.93–1.09]; P=0.86
Phospholipids in large LDL (mmol/L)	0.36±0.095	0.98 [0.90–1.07]; P=0.64
Cholesterol esters in large LDL (mmol/L)	0.77±0.25	0.94 [0.87–1.02]; P=0.15
Total lipids in large LDL (mmol/L)	1.5±0.46	0.97 [0.89–1.05]; P=0.47
Concentration of large LDL particles (nmol/L)	164±51	0.98 [0.90–1.06]; P=0.59

Total cholesterol in medium LDL (mmol/L)	0.60±0.21	0.92 [0.85–1.00]; P=0.05
Phospholipids in medium LDL (mmol/L)	0.24±0.058	0.87 [0.80–0.95]; P=0.001
Cholesterol esters in medium LDL (mmol/L)	0.44±0.17	0.92 [0.85–1.00]; P=0.05
Total lipids in medium LDL (mmol/L)	0.88±0.29	0.92 [0.84–1.00]; P=0.05
Concentration of medium LDL particles (nmol/L)	134±44	0.92 [0.84–1.00]; P=0.05
Total cholesterol in small LDL (mmol/L)	0.36±0.13	0.92 [0.85–1.00]; P=0.05
Total lipids in small LDL (mmol/L)	0.57±0.19	0.92 [0.84–1.00]; P=0.05
Concentration of small LDL particles (nmol/L)	155±51	0.90 [0.82–0.98]; P=0.01
Total cholesterol in very large HDL (mmol/L)	0.18±0.11	0.88 [0.81–0.97]; P=0.01
Free cholesterol in very large HDL (mmol/L)	0.060±0.031	0.94 [0.85–1.03]; P=0.17
Phospholipids in very large HDL (mmol/L)	0.24±0.13	1.07 [0.98–1.17]; P=0.11
Triglycerides in very large HDL (mmol/L)	0.016±0.0067	1.02 [0.93–1.11]; P=0.73
Cholesterol esters in very large HDL (mmol/L)	0.13±0.076	0.86 [0.78–0.94]; P=0.002
Total lipids in very large HDL (mmol/L)	0.43±0.24	0.97 [0.89–1.07]; P=0.55
Concentration of very large HDL particles (nmol/L)	365±205	1.01 [0.92–1.11]; P=0.83
Total cholesterol in large HDL (mmol/L)	0.45±0.23	0.97 [0.89–1.07]; P=0.57
Free cholesterol in large HDL (mmol/L)	0.10±0.054	1.03 [0.94–1.13]; P=0.50
Phospholipids in large HDL (mmol/L)	0.44±0.19	1.04 [0.95–1.13]; P=0.41
Cholesterol esters in large HDL (mmol/L)	0.36±0.17	0.96 [0.87–1.05]; P=0.33
Total lipids in large HDL (mmol/L)	0.93±0.42	1.01 [0.93–1.11]; P=0.75
Concentration of large HDL particles (nmol/L)	1200±530	1.03 [0.94–1.13]; P=0.53
Total cholesterol in medium HDL (mmol/L)	0.56±0.1	0.89 [0.81–0.97]; P=0.01
Free cholesterol in medium HDL (mmol/L)	0.10±0.022	0.90 [0.82–0.98]; P=0.02
Phospholipids in medium HDL (mmol/L)	0.47±0.084	0.91 [0.82–1.00]; P=0.06
Cholesterol esters in medium HDL (mmol/L)	0.46±0.084	0.88 [0.80–0.97]; P=0.006
Total lipids in medium HDL (mmol/L)	1.1±0.18	0.90 [0.81–0.99]; P=0.03
Concentration of medium HDL particles (nmol/L)	1950±340	0.91 [0.82–1.00]; P=0.05
Triglycerides in small HDL (mmol/L)	0.045±0.022	1.01 [0.93–1.10]; P=0.85
Total lipids in small HDL (mmol/L)	1.2±0.13	0.75 [0.68–0.82]; $P=1\times10^{-9}$
Concentration of small HDL particles (nmol/L)	4670±420	0.70 [0.64–0.77]; $P=3\times10^{-14}$
**Composite lipid measures**		
Total triglycerides (mmol/L)	1.6±1.0	0.93 [0.85–1.01]; P=0.09
Triglycerides in chylomicrons and extremely large VLDL (mmol/L)	0.022±0.030	0.96 [0.88–1.05]; P=0.37
Triglycerides in VLDL (mmol/L)	1.1±0.9	0.91 [0.84–1.00]; P=0.05
Triglycerides in IDL (mmol/L)	0.12±0.05	1.05 [0.97–1.14]; P=0.22
Total cholesterol (mmol/L)	5.4±1.1	0.91 [0.84–0.99]; P=0.02
Total cholesterol in IDL (mmol/L)	0.79±0.22	1.01 [0.94–1.10]; P=0.73
Total cholesterol in LDL (mmol/L)	2.0±0.7	0.94 [0.86–1.02]; P=0.13
Total cholesterol in HDL (mmol/L)	1.7±0.4	0.89 [0.81–0.98]; P=0.01
VLDL particle size (mean diameter, nm)	37±1.9	0.84 [0.77–0.92]; P=0.0001
LDL particle size (mean diameter, nm)	24±0.3	1.32 [1.21–1.45]; $P=9\times10^{-10}$
HDL particle size (mean diameter, nm)	9.9±0.3	1.08 [0.99–1.17]; P=0.10
Double bond protons of mobile lipids (cu)	2.9±1.0	0.83 [0.75–0.91]; $P=9\times10^{-5}$
Methylene groups of mobile lipids (cu)	39±21	0.94 [0.86–1.03]; P=0.20
Methyl groups of mobile lipids (cu)	11±3	0.87 [0.79–0.96]; P=0.005

**Proteins**		
Apolipoprotein A-1 [g/L]	1.8±0.2	0.80 [0.73–0.87]; P=1×10^{-7}
Apolipoprotein B [g/L]	1.1±0.3	0.94 [0.86–1.02]; P=0.13
Albumin (cu)	101±8	0.66 [0.61–0.71]; P=2×10^{-27}
Alpha-1-acid glycoprotein (cu)	1.6±0.3	1.34 [1.25–1.43]; P=6×10^{-16}
**Amino acids**		
Alanine (μmol/L)	350±91	0.91 [0.83–1.00]; P=0.04
Glutamine (μmol/L)	380±83	0.83 [0.76–0.90]; P=2×10^{-5}
Histidine (μmol/L)	60±14	0.70 [0.64–0.76]; P=3×10^{-16}
Leucine (μmol/L)	65±19	0.89 [0.81–0.97]; P=0.01
Isoleucine (μmol/L)	52±22	0.87 [0.80–0.95]; P=0.003
Valine (μmol/L)	190±46	0.77 [0.70–0.85]; P=5×10^{-8}
Phenylalanine (μmol/L)	54±10	1.16 [1.08–1.25]; P=0.0001
Tyrosine (μmol/L)	67±17	0.97 [0.89–1.06]; P=0.52
**Miscellaneous Metabolites**		
Glucose (mmol/L)	4.3±1.5	1.15 [1.10–1.22]; P=1×10^{-7}
Lactate (mmol/L)	2.4±0.9	1.11 [1.01–1.21]; P=0.03
Pyruvate (μmol/L)	127±35	1.10 [1.01–1.20]; P=0.02
Citrate (μmol/L)	99±34	1.15 [1.06–1.25]; P=0.0008
beta-hydroxybutyrate (μmol/L)	87±84	1.10 [1.03–1.18]; P=0.005
Acetate (μmol/L)	28±27	1.05 [0.98–1.14]; P=0.18
Acetoacetate (μmol/L)	37±45	1.04 [1.01–1.07]; P=0.02
Creatinine (μmol/L)	62±21	1.07 [1.02–1.12]; P=0.006
Urea (μmol/L)	156±36	0.94 [0.86–1.02]; P=0.15

Mean±SD concentrations and hazard ratios [95% confidence interval] for all-cause mortality in the Estonian Biobank cohort. Hazard ratios are per 1-SD increment in biomarker concentration. Hazard ratios were assessed separately for each biomarker by Cox models adjusted for age and sex. cu: standardized concentration units.

All candidate biomarker concentrations were measured from native plasma or serum by high-throughput NMR spectroscopy. Molecular information on lipoprotein lipid concentrations and particle sizes is reflected in the NMR spectra of plasma and serum [1]. Quantification of lipoprotein lipids by NMR was enabled by regression models calibrated against high-performance liquid chromatography on an external set of samples [2,3]. The 14 lipoprotein subclass sizes were defined as follows: extremely large VLDL with particle diameters from 75 nm upwards and a possible contribution of chylomicrons, five VLDL subclasses (average particle diameters of 64.0 nm, 53.6 nm, 44.5 nm, 36.8 nm, and 31.3 nm), IDL (28.6 nm), three LDL subclasses (25.5 nm, 23.0 nm, and 18.7 nm), and four HDL subclasses (14.3 nm, 12.1 nm, 10.9 nm, and 8.7 nm) [3]. The mean size for VLDL, LDL and HDL particles was calculated by weighting the corresponding subclass diameters with their particle concentrations. For instance, the mean diameter of VLDL particles is calculated as $D_{\mathrm{VLDL}} = \sum_i d_i \cdot p_i / \sum_i p_i$, where the summation runs from very small VLDL to extremely large VLDL, d_i are the particle diameters listed above and p_i the corresponding VLDL particle concentrations.

Concentrations of certain abundant proteins can also be quantified by NMR [2]. For instance, albumin is so abundant in blood that it leaves a background signal on the NMR spectrum, which is directly proportional to the concentration. Alpha-1-acid glycoprotein gives rise to a characteristic glycoprotein acetyl spike in the NMR spectrum that is proportional to the concentration. The measurement of alpha-1-acid glycoprotein may contain minor influences of other glycoproteins, however, this additional variability is unlikely to inflate risk estimates [4]. Similarly, the

concentration of citrate can be quantified from its signal areas, which become visible when signals from the lipid molecules are suppressed by spectroscopic settings. Quantification in µmol/L was enabled by regression modelling with a reference of known concentration.

The coefficients of variations for the 4 highlighted biomarkers were 1.4 % for alpha-1-acid glycoprotein, 1.1% for albumin, 1.1 %, 0.4 % for VLDL particle size, and 3.5 % for citrate.

1. Mallol R, Rodriguez MA, Brezmes J, Masana L, Correig X (2013) Human serum/plasma lipoprotein analysis by NMR: Application to the study of diabetic dyslipidemia. Prog Nucl Magn Reson Spectrosc 79:1–24
2. Soininen P, Kangas AJ, Würtz P, Tukianen T, Tynkkynen T, et al (2009) High-throughput serum NMR metabonomics for cost-effective holistic studies on systemic metabolism. Analyst 134:1781–1785.
3. Okazaki M, Usui S, Ishigami M, Sakai N, Nakamura T, Matsuzawa Y, Yamashita S (2005) Identification of unique lipoprotein subclasses for visceral obesity by component analysis of cholesterol profile in high-performance liquid chromatography. Arterioscler Thromb Vasc Biol 25:578–584.
4. Nicholson JK, Foxall PJD, Spraul M, Farrant RD, Lindon JC (1995) 750-MHz ^{1}H and ^{1}H-^{13}C NMR spectroscopy of human blood plasma. *Anal Chem* 67:793–811
