# Supplementary material for: Biomarker Profiling by Nuclear Magnetic Resonance Spectroscopy for the Prediction of All-Cause Mortality: An Observational Study of 17,345 Persons
Source: PLoS Med. 2014 Feb 25;11(2):e1001606. doi: 10.1371/journal.pmed.1001606 (PMC3934819; doi:10.1371/journal.pmed.1001606)
Supplement: Table S2 — Reclassification tables of individuals who died and who did not die during 5 y of follow up in the FINRISK cohort. (PDF) [file pmed.1001606.s007.pdf]

Table S2. Reclassification of individuals who died and who did not die during 5 y of follow-up in the FINRISK cohort.

Individuals Who Died		Risk Score with Established Risk Factors and Biomarkers			
		< 1.25%	1.25–2.50%	2.5%–5.0%	> 5.0%
Reference Risk Score with Established Risk Factors	< 1.25%	12 (50%)	8 (33%)	3 (13%)	1 (4%)
	1.25–2.50%	6 (22%)	8 (30%)	8 (30%)	5 (19%)
	2.5%–5.0%	0 (0%)	9 (26%)	12 (35%)	13 (38%)
	> 5.0%	0 (0%)	3 (4%)	16 (22%)	55 (74%)
**Reclassification improvement for participants who died: 2.5±5.3%; P=0.64**					
**Individuals Who Did Not Die**		**Risk Score with Established Risk Factors and Biomarkers**			
		< 1.25%	1.25–2.50%	2.5%–5.0%	> 5.0%
Reference Risk Score with Established Risk Factors	< 1.25%	3667 (90%)	309 (8%)	77 (2%)	17 (0%)
	1.25–2.50%	543 (44%)	465 (37%)	185 (15%)	52 (4%)
	2.5%–5.0%	81 (11%)	274 (34%)	293 (37%)	141 (18%)
	> 5.0%	6 (1%)	83 (11%)	243 (32%)	425 (56%)
**Reclassification improvement for participants who did not die: 6.7±0.7%; $P=2\times10^{-24}$**					
**Net reclassification improvement: 9.2±5.4%**					

Reclassifation was examined in the FINRISK validation cohort based on the risk prediction scores derived in the Estonian Biobank cohort (Supplementary Table S2). The established risk factors in the reference risk prediction score were age, sex, body mass index, systolic blood pressure, fasting time, total cholesterol, high-density lipoprotein cholesterol, triglycerides, creatinine, smoking, alcohol consumption, prevalent diabetes, prevalent cardiovascular disease, and prevalent cancer. The biomarker risk prediction score was extended with alpha-1-acid glycoprotein, albumin, very-low-density lipoprotein particle size, and citrate.

Each cell indicates the number of persons classified to be in the risk category based on the two risk prediction scores. The fraction, with respect to the reference score classifcation, is shown in parentheses. For example, the top right corner cell indicates that 1 person, who died during 5 y follow-up, was classified with risk <1.25% according to the reference score, but with risk >5.0% according to the risk prediction score extended with the biomarkers. Similarly, 243 persons, who did not die during 5 y follow-up, were classified with >5.0% risk based on the reference score, but correctly down-classified to 2.5-5.0% based on the risk prediction score with biomarkers. The 243 persons correspond to 32% of the persons classified with >5.0% risk based on the reference score. Similarly, 141 persons were incorrectly up-classified in risk from 2.5-5.0% to >5.0 based on the risk prediction score with novel biomarkers.
