# Supplementary material for: Biomarker Profiling by Nuclear Magnetic Resonance Spectroscopy for the Prediction of All-Cause Mortality: An Observational Study of 17,345 Persons
Source: PLoS Med. 2014 Feb 25;11(2):e1001606. doi: 10.1371/journal.pmed.1001606 (PMC3934819; doi:10.1371/journal.pmed.1001606)
Supplement: Table S3 — Hazard ratios for all-cause mortality in the Estonian Biobank cohort stratified by gender. (PDF) [file pmed.1001606.s008.pdf]

Table S3. Hazard ratios for all-cause mortality in the Estonian Biobank cohort stratified by gender.

**Biomarker**	**Men**<br><br>**Hazard ratio (95% CI); P**	**Women**<br><br>**Hazard ratio (95% CI); P**
Alpha-1-acid glycoprotein	1.62 (1.44-1.82) $P=9\times10^{-16}$	1.77 (1.54-2.04) $P=9\times10^{-16}$
Albumin	0.67 (0.60-0.74) $P=3\times10^{-13}$	0.73 (0.64-0.83) $P=4\times10^{-6}$
VLDL particle size	0.64 (0.56-0.73) $P=2\times10^{-10}$	0.67 (0.56-0.79) $P=3\times10^{-6}$
Citrate	1.53 (1.33-1.76) $P=2\times10^{-9}$	1.21 (1.06-1.38) P=0.004

Hazard ratios (95% confidence intervals) are per 1-SD increment in biomarker concentration, and adjusted for age as time-scale, HDL cholesterol, current smoking, prevalent diabetes, prevalent cardiovascular disease, and prevalent cancer.
